# Expression and Functional Characterization of the *Agrobacterium* VirB2 Amino Acid Substitution Variants in T-pilus Biogenesis, Virulence, and Transient Transformation Efficiency

**DOI:** 10.1371/journal.pone.0101142

**Published:** 2014-06-27

**Authors:** Hung-Yi Wu, Chao-Ying Chen, Erh-Min Lai

**Affiliations:** 1 Institute of Plant and Microbial Biology, Academia Sinica, Taipei, Taiwan; 2 Department of Plant Pathology and Microbiology, National Taiwan University, Taipei, Taiwan; University of Wisconsin-Milwaukee, United States of America

## Abstract

*Agrobacterium tumefaciens* is a phytopathogenic bacterium that causes crown gall disease by transferring transferred DNA (T-DNA) into the plant genome. The translocation process is mediated by the type IV secretion system (T4SS) consisting of the VirD4 coupling protein and 11 VirB proteins (VirB1 to VirB11). All VirB proteins are required for the production of T-pilus, which consists of processed VirB2 (T-pilin) and VirB5 as major and minor subunits, respectively. VirB2 is an essential component of T4SS, but the roles of VirB2 and the assembled T-pilus in *Agrobacterium* virulence and the T-DNA transfer process remain unknown. Here, we generated 34 VirB2 amino acid substitution variants to study the functions of VirB2 involved in VirB2 stability, extracellular VirB2/T-pilus production and virulence of *A. tumefaciens*. From the capacity for extracellular VirB2 production (ExB2^+^ or ExB2^−^) and tumorigenesis on tomato stems (Vir^+^ or Vir^−^), the mutants could be classified into three groups: ExB2^−^/Vir^−^, ExB2^−^/Vir^+^, and ExB2^+^/Vir^+^. We also confirmed by electron microscopy that five ExB2^−^/Vir^+^ mutants exhibited a wild-type level of virulence with their deficiency in T-pilus formation. Interestingly, although the five T-pilus^−^/Vir^+^ uncoupling mutants retained a wild-type level of tumorigenesis efficiency on tomato stems and/or potato tuber discs, their transient transformation efficiency in *Arabidopsis* seedlings was highly attenuated. In conclusion, we have provided evidence for a role of T-pilus in *Agrobacterium* transformation process and have identified the domains and amino acid residues critical for VirB2 stability, T-pilus biogenesis, tumorigenesis, and transient transformation efficiency.

## Introduction

*Agrobacterium tumefaciens* is a Gram-negative plant pathogenic bacterium that causes crown gall disease in a wide range of plants [1]. *A. tumefaciens* can sense plant-released phenolic compounds (e.g., acetosyringone; AS) to activate the expression of virulence factors for infection. The VirA/VirG two-component system is responsible for the phenolics-induced virulence (*vir*) gene expression [2]. Transfer DNA (T-DNA) located on the tumor-inducing (Ti) plasmid is recognized and processed by the VirD1/VirD2 relaxosome-like protein complex and covalently linked with the VirD2 protein to form the T-strand. The T-DNA and effector protein substrates are transferred through the VirB/VirD4 assembled type IV secretion system (T4SS) into host plant cells [2]–[4].

The *A. tumefaciens* VirB/VirD4 T4SS consists of the envelope-spanning translocation channel and the extracellular T-pilus structure [5]–[8]. Accumulating biochemical and genetic data suggest a possible VirB/D4 T4SS assembly and T-DNA translocation pathway [9], [10]. The T-DNA immunoprecipitation (TrIP) technique revealed that T-DNA was first recruited by the VirD4 coupling protein, then the substrate passed to the inner-membrane–associated ATPase VirB11. The VirD4/VirB4 ATPases provide energy for T-DNA transfer to the inner-membrane proteins VirB6/VirB8 followed by passage to VirB2/VirB9 presumably localized at the distal end of the T4SS transmembrane complex [10]. Recent cryo-electron microscopy (cryo-EM) and crystallographic structure studies of the *Escherichia coli* conjugative plasmid pKM101 revealed that the T4SS core complex consisted of 14 copies of each of the VirB7-like TraN, VirB9-like TraO, and VirB10-like TraF subunits forming two layers of a double-walled ring structure inserted in the inner and outer membranes [11], [12]. Remarkably, eight T4SS proteins (VirB3–VirB11) encoded by the R388 conjugative plasmid can assemble into an approximately 3-MDa nanomachine spanning the double membranes, which was visualized and reconstructed by electron microscopy [13]. VirB10 may function dynamically to couple cytoplasmic-membrane ATPases with ATP energy to gate the outer-membrane translocation channel via a conformational switch. Interestingly, VirD4 coupling protein not only functions as a receptor for protein substrates [14]; a recent study revealed that DNA but not protein binding to VirD4 and VirB11 activates the VirB10 structural transition and enables DNA transfer [14]. This study also suggested that translocation of DNA and protein substrates through T4SS may be mechanistically distinct processes [14].

*Agrobacterium* T-pilus is composed of the major subunit VirB2 and the minor component VirB5 [7], [15], [16]. All VirB proteins (VirB1 to VirB11) but not the VirD4 coupling protein is essential for T-pilus biogenesis [6]. Pilin subunits typically undergo an additional post-translational modification reaction after removal of its N-terminal signal peptide, including acetylation of F-like pilin or cyclization of P-like pilin and T-pilin [17]–[20]. The 12.3-kDa VirB2 precursor is processed into a 7.2-kDa product by removal of a long N-terminal signal peptide in both *E. coli* and *A. tumefaciens*, but the cyclization occurs only in *A. tumefaciens* in a Ti-plasmid–independent manner [21]. Consistent with predicted topology [19], experimental evidence revealed that processed VirB2 consists of two hydrophobic trans-membrane domains linked by an intervening hydrophilic loop (residues 90–94) inside the cytoplasm and by a periplasmic loop formed by linkage between hydrophilic N- and C-termini at residues 48 and 121 [22].

In contrast to much better-defined functions for the T4SS translocation channel, the roles of T4SS extracellular pilus remain obscure. T4SS substrate translocation from bacteria into host cells likely requires close contact with target cells [23] and pili may play a role during this process. A recent study revealed that the *A. tumefaciens* VirB/D4 T4SS forms helically arranged foci around the bacterial cell that may help maximize effective contact and transfer of substrate to host cells [24]–[26]. Therefore, T-pili may help *A. tumefaciens* bind to plant cells for close contact [25]. Alternatively, pili may also provide a channel for substrate translocation through their hollow lumen [27], [28]. The transfer of DNA via the T4SS pili could be observed by direct visualization of low-efficiency conjugal transfer events via *E. coli* F-pili when cells were separated up to 1.2 µm [29]. Furthermore, DNA could be detected in the F-pilus channels during conjugation [30] and the *Helicobacter pylori* T4SS substrate protein CagA could be detected at the tip of the pilus [31]. However, mutants that block biogenesis of the T-pilus but not substrate transfer could be isolated by amino acid substitution in several T4SS components such as VirB6, VirB9, VirB10 and VirB11 of *A. tumefaciens* [14], [32]–[36]. Because VirB2 protein is a T4SS component required for substrate translocation, isolation of these “uncoupling” mutants suggested that intracellular VirB2 but not its assembled T-pilus is required for T4SS-mediated T-DNA/effector translocation. Thus, the role of T-pilus remains unknown.

In this study, we used site-directed mutagenesis to generate various VirB2 single amino acid substitution variants to identify the amino acid residues critical for VirB2 stability, extracellular VirB2/T-pilus production, and virulence. Notably, we isolated five T-pilus^−^/Vir^+^ uncoupling mutants that retain a wild-type level of tumorigenesis efficiency on tomato stems and/or potato tuber discs but are highly attenuated in transient transformation efficiency in *Arabidopsis* seedlings. These data suggest a role of T-pilus in the *Agrobacterium* transformation process.

## Results

### VirB2 family proteins comprise variable N-terminal signal peptides and conserved C-terminal processing products

**Figure 1:**
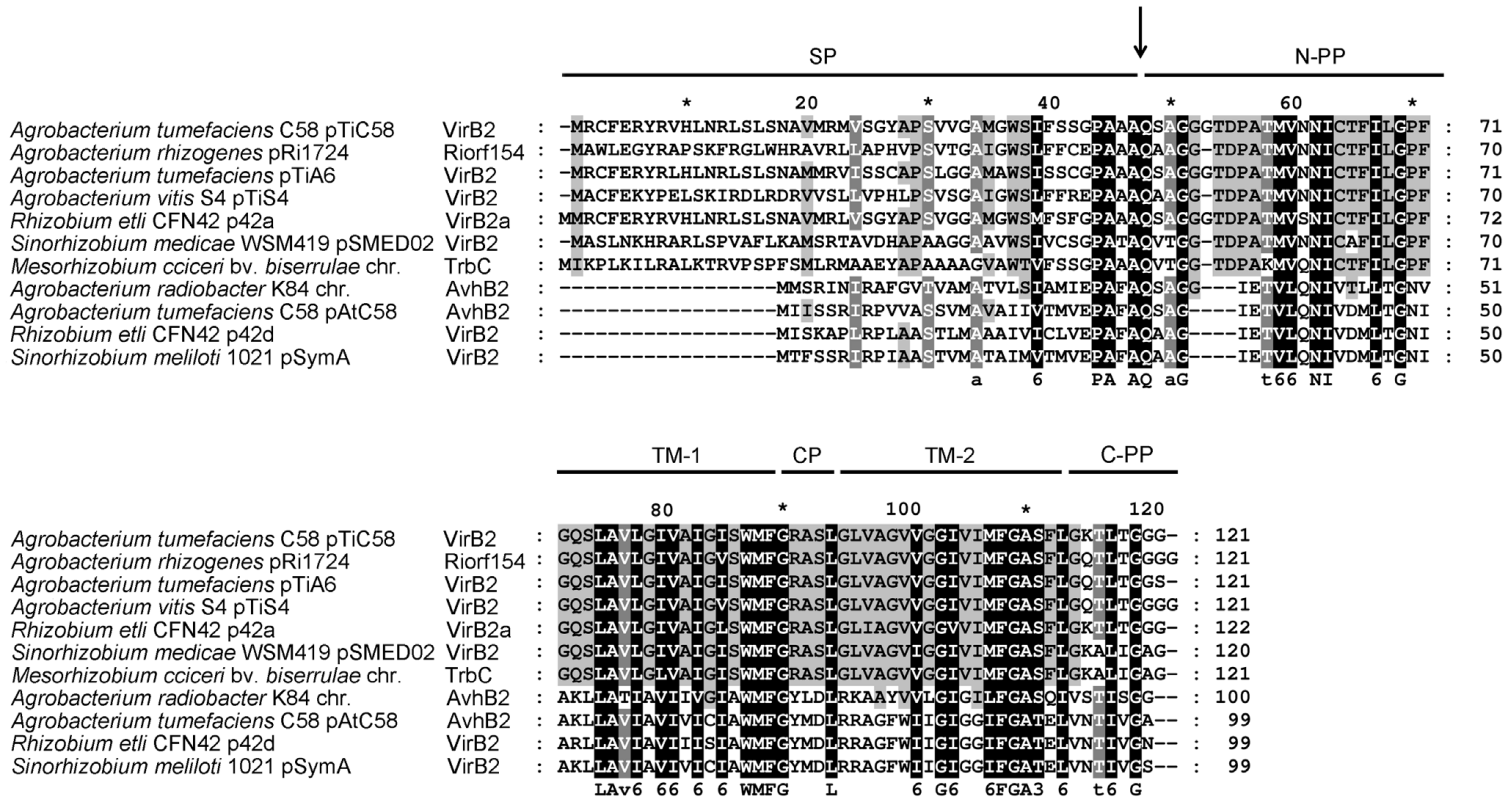
Amino acid sequence alignment of VirB2 family proteins. Multiple amino acid sequence alignment of VirB2 homologues with ClustalW2 [50]. The organism/plasmid name for each homolog is indicated on the left of the aligned sequence. UniProt accession numbers: *Agrobacterium tumefaciens* pTiC58, P17792; *Agrobacterium rhizogenes* pRi1724, Q9F5A1; *Agrobacterium tumefaciens* pTiA6, P05351; *Agrobacterium vitis* pTiS4, B9K417; *Rhizobium etli* p42a, Q2K2L1; *Sinorhizobium medicae* pSMED02; A6UMA7, *Mesorhizobium ciceri* chromosome (chr.), E8TGI0; *Agrobacterium radiobacter* K84 chromosome (chr.), B9JE70; *Agrobacterium tumefaciens* pAtC58, Q7D3S1; *Rhizobium etli* p42d, Q8KIM6; *Sinorhizobium meliloti* pSymA, Q92YZ4. The amino acid residues identical in all proteins are in black and those conserved in most but not all of the proteins are in gray. The arrow indicates the processing site of VirB2 encoded by pTiC58. Each region/domain of VirB2 is indicated: SP, signal peptide; TM-1, trans-membrane domain 1; CP, cytoplasmic domain; TM-2, trans-membrane domain 2; N-PP, N-terminal periplasmic domain; C-PP, C-terminal periplasmic domain.

To determine the amino acid residues critical for the function of VirB2 in *Agrobacterium* virulence and T-pilus production, we first compared the amino acid sequences of VirB2 homologs encoded by various agrobacteria and rhizobia (Figure 1). The N-terminal signal peptide of VirB2 is variable, but the mature processed T-pilin region (including periplasmic, trans-membrane and cytoplasmic domains) is highly conserved (Figure 1 and Table S1). A conserved motif PAxAQ at the processing site is critical for precise signal peptide removal and cyclization of processed T-pilin [17], [21].

### Identification of domains and amino acid residues critical for VirB2 stability, processing, and extracellular VirB2 production

**Figure 2:**
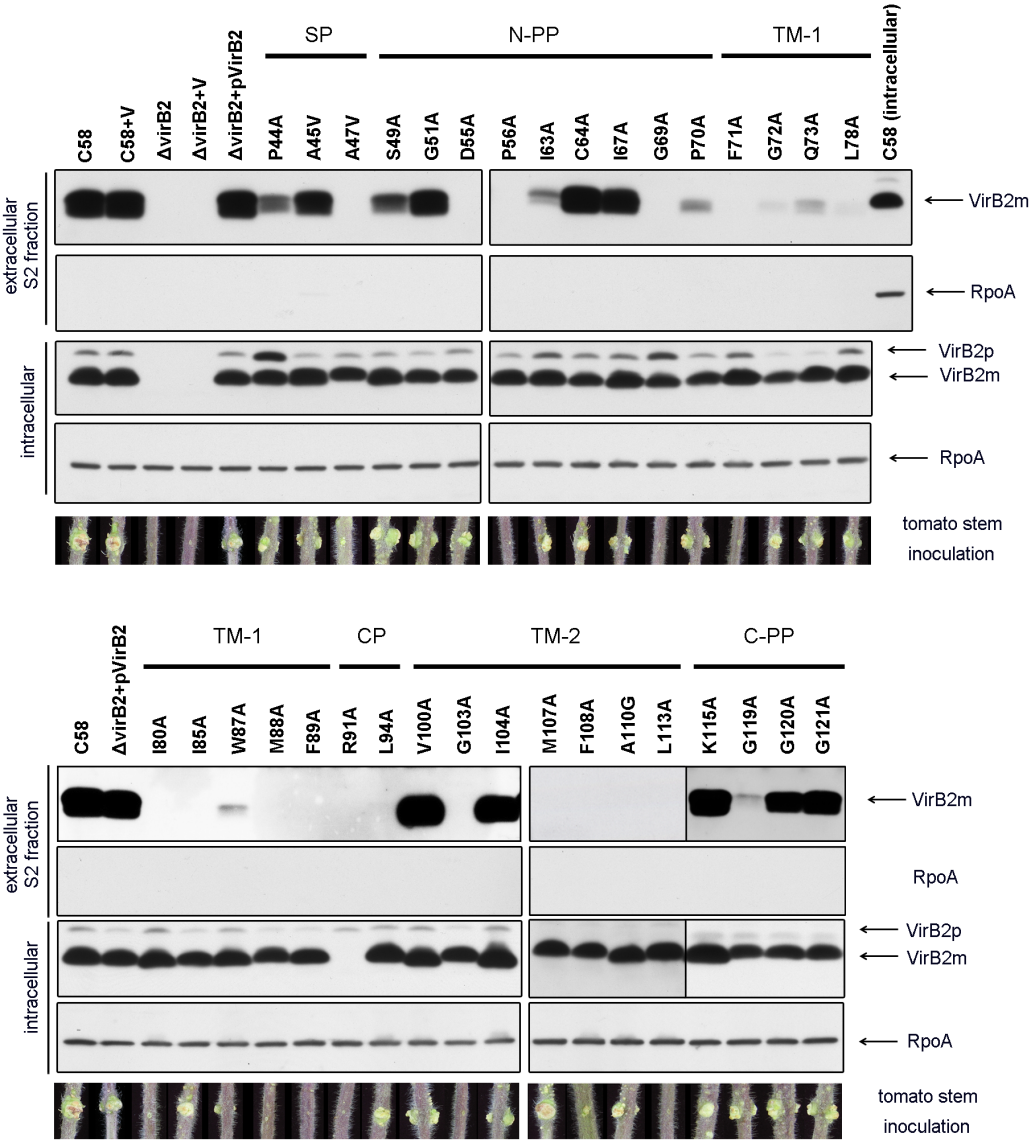
Western blot analysis of the intracellular and extracellular S2 fractions, and tumor assays on tomato stem of VirB2 variants. *A. tumefaciens* cells grown on acetosyringone (AS)-induced AB-MES (pH 5.5) agar at 19°C for 3 days [7] were collected to isolate intracellular proteins and extracellular S2 fractions. C58, *A. tumefaciens* wild type strain C58; V, empty vector pRL662; Δ*virB2*, *virB2* deletion mutant; Δ*virB2*(pVirB2), expression of wild type *virB*p-*B1*-*B2*-*B3* in Δ*virB2*. Western blot analysis with antisera against VirB2 B24 peptide or B23 peptide (for variants in C-PP) or RNA polymerase RpoA, as an internal control. Unprocessed VirB2 precursor is indicated as VirB2p and processed mature VirB2 as VirB2m. Shows representative tumor assay results on tomato stems. Similar results were obtained from at least three independent experiments (3–5 plants for each mutant in each independent experiment). Each region/domain of VirB2 is indicated as described in Figure 1.

For full complementation of *Agrobacterium* virulence in a *virB2* in-frame deletion mutant, *virB2* must be co-expressed with adjacent genes and driven by its native promoter [37]. Therefore, we cloned the DNA fragment containing the *virB* promoter and *virB1*, *virB2* and *virB3* genes (*virB*p-*B1*-*B2*-*B3*) into a broad host-range plasmid, pRL662, for expression of wild-type VirB2 and all VirB2 variants in the *virB2* in-frame deletion mutant (Δ*virB2*) derived from the *A. tumefaciens* wild-type C58 strain. The conserved or non-conserved amino acid residues near the processing site or different domains within the T-pilin region were randomly chosen for substitution with Alanine (A). *A. tumefaciens* cells induced for T-pilus production were scraped off the agar plate, resuspended in acidic phosphate buffer (pH 5.3), and centrifuged to obtain the S1 fraction. Cell pellets were resuspended again and subjected to shearing to obtain the S2 fraction enriched for T-pilus. Both intracellular and extracellular VirB2 levels were restored to wild-type levels in Δ*virB2* complemented with wild-type VirB2 (pVirB2), which was consistent with its full complementation by tumorigenesis analysis on tomato stems (Figure 2). Consistent with our previous study [21], we detected both pro-pilin (12.3-kDa unprocessed VirB2 precursor, named VirB2p) and T-pilin (7.2-kDa processed mature VirB2, named VirB2m) inside cells, but only processed T-pilin was detected extracellularly (Figure 2, also see Figure S1 for lower intensity of western blot signals). Extracellular VirB2 was more abundant in the S2 than S1 fraction, and all variants with no detectable VirB2 in the S2 fraction also did not produce any VirB2 signals in the S1 fraction (Figure 2 and S2). Because the detection of VirB2 in the sheared S2 fraction by western blot analysis agrees with the observation of T-pilus by electron microscopy and vice versa [6], we used western blot analysis of the S2 fraction as a first step to screen the mutant phenotype for T-pilus production.

For all five VirB2 variants with amino acid substitutions near the processing site (from P44 to G51), we detected both VirB2p and wild-type levels of VirB2m within the cells (Figure 2). Notably, extracellular VirB2 levels were reduced from the P44A and S49A variants and not detected from the A47V variant. Increased intracellular VirB2p from the P44A variant suggested a role of P44 for processing efficiency. The absence of extracellular VirB2 and slower migration of the intracellular VirB2m from the A47V variant relative to wild-type VirB2m implied that the A47V variant may undergo incorrect processing or cyclization, thus leading to defects in production of extracellular VirB2 or T-pilus. Strikingly, all variants except the R91A variant accumulated comparable intracellular VirB2m levels, in which only the unprocessed but not processed R91A variant could be detected (Figure 2). Because R91 is the sole positively charged residue within the cytoplasmic domain, substitution of Arginine 91 with Alanine may break the “positive inside rule” [38] and result in the instability of the R91A variant after processing.

**Figure 3:**
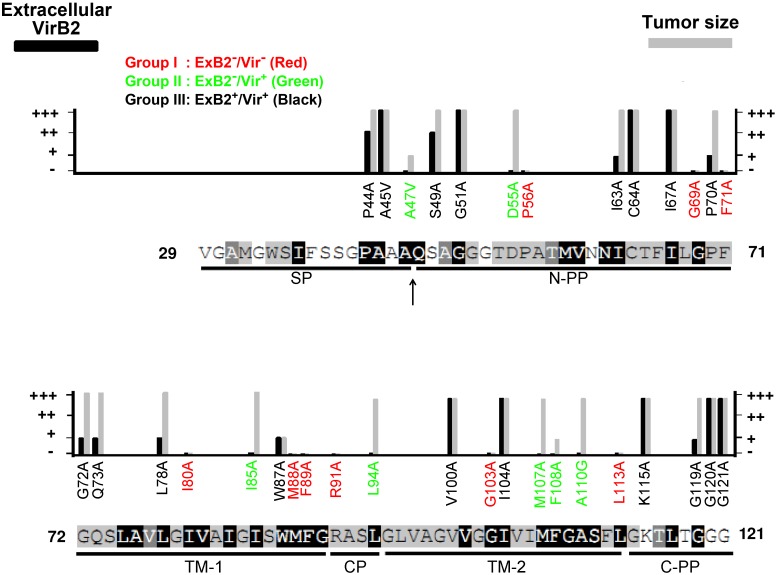
Phenotype summary of VirB2 variants. VirB2 amino acid substitutions are indicated with the levels of extracellular VirB2 (ExB2) production and occurrence or size of tumor formation on tomato stems (Vir) with wild type (+++), modest reduction (++), highly attenuation (+), or loss (−). VirB2 protein sequences with indicated conserved amino acid residues, regions/domains, and the processing site (indicated by an arrow) are presented as described in Figure 1. The 34 VirB2 variants are classified into three groups: ExB2^−^/Vir^−^, ExB2^−^/Vir^+^, and ExB2^+^/Vir^+^ shown in red, green and black, respectively.

Little or no extracellular VirB2 could be detected in all the mutants with amino acid substitutions in trans-membrane domain 1 (TM1) (Figure 3). In contrast, substitutions located in the N-terminal periplasmic domain (N-PP), trans-membrane domain 2 (TM2), and C-terminal periplasmic domain (C-PP) had differential effects on the accumulation of extracellular VirB2m. Therefore, these domains of processed VirB2 are all indispensable, but the integrity of the TM1 and its adjacent regions are most critical for production of extracellular VirB2m.

### Identification of VirB2 variants uncoupling virulence and T-pilus biogenesis phenotypes

Tumorigenicity of each VirB2 variant was first determined by infection on tomato stems, and their virulence was evaluated by the occurrence or size of the tumors formed. From the capacity for extracellular VirB2 production (ExB2^+^ or ExB2^−^) and tumorigenesis on tomato stems (Vir^+^ or Vir^−^), the mutants were classified into three groups: ExB2^−^/Vir^−^, ExB2^−^/Vir^+^, and ExB2^+^/Vir^+^ (Figure 3). Although the combination of extracellular VirB2 production and virulence phenotypes theoretically can result in four groups of mutant phenotypes, we identified only three groups from our mutant pools. Group I mutants with the ExB2^−^/Vir^−^ phenotype led us to identify the amino acid residues crucial for both extracellular VirB2 production and tumorigenesis. These amino acid residues are mostly dispersed across all domains within the T-pilin region, but three (M88A, F89A, R91A) are located at the junction of TM1 and cytoplasmic domain (CP). Group II mutants with the ExB2^−^/Vir^+^ phenotype showed the amino acids residues critical for extracellular VirB2 production but dispensable for tumorigenesis. Strikingly, all mutants defective in virulence also lost extracellular VirB2 production (group I mutants) and all mutants capable of extracellular VirB2 production were virulent (group III mutants). These VirB2 amino acid residues required for *A. tumefaciens* to form a functional T4SS for substrate transfer may also be essential for production of extracellular VirB2.

**Figure 4:**
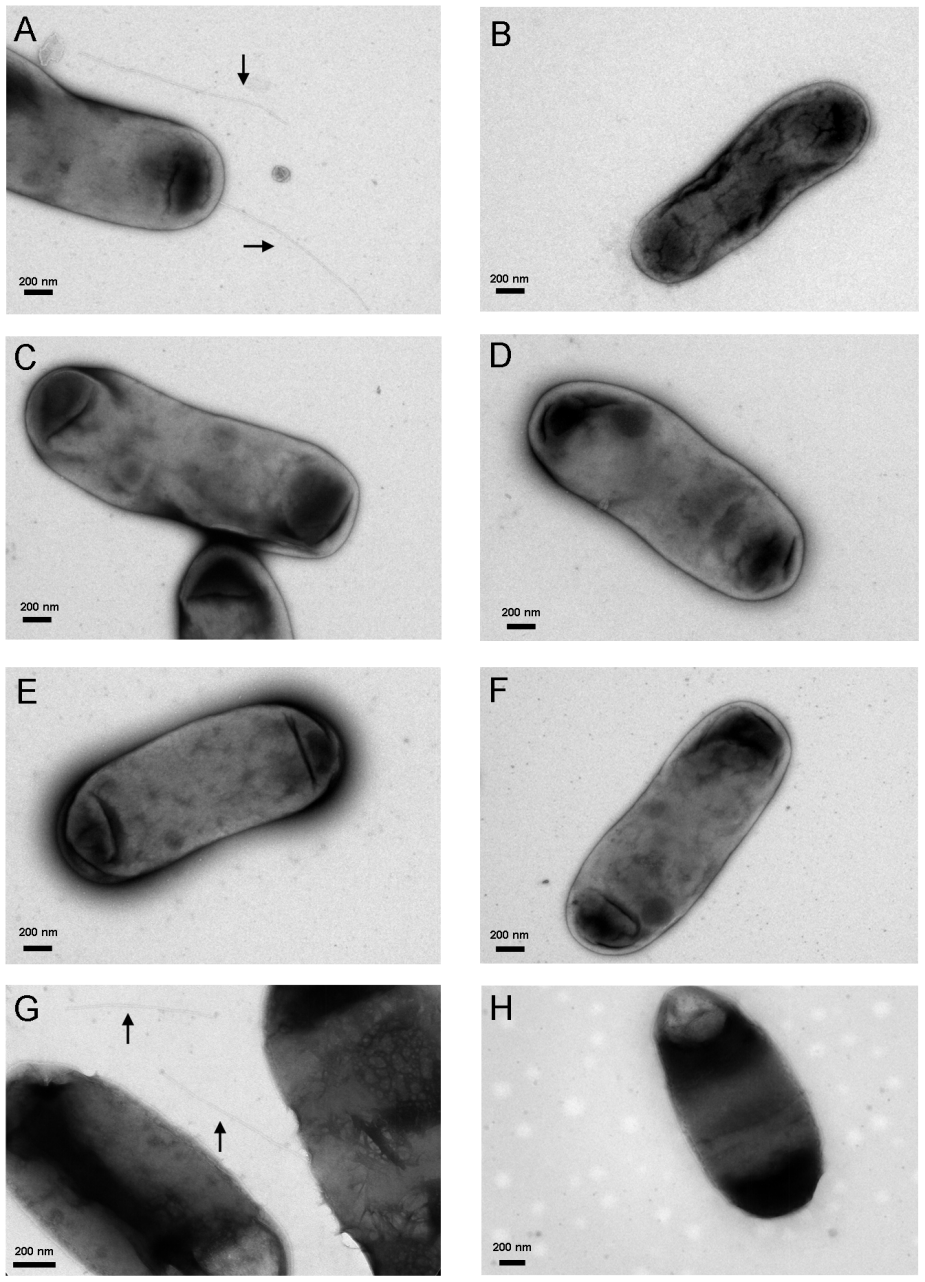
T-pilus observation by transmission electron microscopy (TEM). *A. tumefaciens* cells grown on T-pilus induction condition were collected and stained with 2% uranyl acetate to visualize T-pilus by TEM. Shows representative TEM image of *A. tumefaciens* strains producing wild-type VirB2 (A) or VirB2 variants D55A (B), I85A (C), L94A (D), M107A (E), A110G (F), G119A (G) and G119C (H). The rigid, long T-pilus is indicated by an arrow (A, G). Scale bar: 200 nm. All samples were examined for T-pilus formation by examining hundreds of bacterial cells from at least two independent experiments.

Among the three groups of mutants, we were particularly interested in the five variants classified in the ExB2^−^/Vir^+^ group because these VirB2 variants (D55A, I85A, L94A, M107A, A110G) can incite the wild-type size of tumors on tomato stem but did not produce detectable extracellular VirB2. Although the loss of extracellular VirB2 production agrees with the loss of T-pilus biogenesis [6], we could not exclude that the failure to detect extracellular VirB2 may be due to the formation of short or structurally distinct T-pilus recalcitrant to isolation by shearing from the mutants. Thus, we negatively stained the *A. tumefaciens* cells expressing wild-type VirB2 and these five ExB2^−^/Vir^+^ variants by uranyl acetate and examined them by transmission electron microscopy (TEM) to observe the bacterial cells and associated surface structures. Similar to previous reports [6], [7], [17], we observed T-pilus as a rigid or semi-rigid long filament (500 nm to2 µm) ∼10-nm wide in the wild-type complemented strain Δ*virB2*(pVirB2) in an AS-induction–dependent manner (Figure 4A). In contrast, no T-pilus-like filament could be detected from these ExB2^−^/Vir^+^ mutants (Figure 4B–F). These results agree with the lack of extracellular VirB2 detected by western blot analysis and suggest that these five mutants represent an uncoupling phenotype of virulence and T-pilus biogenesis (Figure 2). Thus, we defined the *A. tumefaciens* strains expressing these five VirB2 variants (D55A, I85A, L94A, M107A, A110G) as T-pilus^−^/Vir^+^ uncoupling mutants.

### T-pilus^−^/Vir^+^ uncoupling mutants show highly attenuated transient transformation efficiency in *Arabidopsis* seedlings

**Figure 5:**
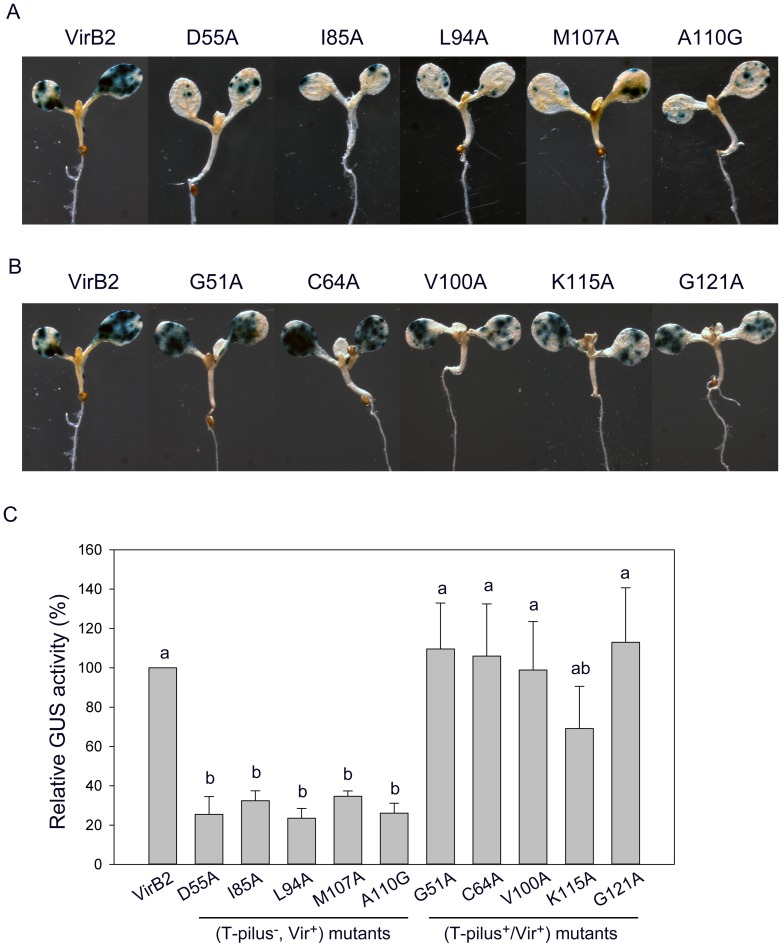
T-pilus^−^/Vir^+^ uncoupling mutants show highly attenuated transient transformation efficiency in *Arabidopsis* seedlings. *A. tumefaciens* strains expressing the wild type or variants of VirB2 harboring the T-DNA vector pBISN1 were used to infect 4-day-old *Arabidopsis* seedlings. GUS activity as a reporter of transient transformation efficiency was determined by GUS staining (A and B) or quantitative activity assay (C) at 3 dpi. (A) GUS staining of T-pilus^−^/Vir^+^ uncoupling mutants and (B) T-pilus^+^/Vir^+^ mutants. (C) Quantitative GUS activity of all mutants. Data are mean±SD of 4 biological repeats from 2 independent experiments (10 seedlings in each biological repeat). The data were analyzed by ANOVA for statistical classification, which revealed two groups (groups a and b) of strains differing in transient GUS activity.

The evidence that the T-pilus^−^/Vir^+^ uncoupling mutant retains the ability to incite tumors on tomato stems suggested that the T-pilus may not be essential for virulence. To test whether these T-pilus^−^/Vir^+^ uncoupling mutants may quantitatively affect *Agrobacterium* virulence or transformation efficiency, we first used the quantitative tumor assay on potato tuber discs to determine their virulence. Two T-pilus^−^/Vir^+^ uncoupling mutants (L94A and A110G) and a randomly selected ExB2^+^/Vir^+^ mutant (G121A) all retained the wild-type tumorigenesis efficiency when infected with 10^6^ or 10^4^ cells (Figure S3). Because tumorigenesis is a complex and long process that may not be sensitive enough to detect quantitative differences in T-DNA transfer efficiency, we adapted a transient transformation assay recently developed in our laboratory [39] for further analysis. This system used the T-DNA–encoded β-glucuronidase *gusA* (*GUS*) gene as a reporter to quantitatively monitor transient transformation efficiency in *Arabidopsis* seedlings. The T-DNA vector pBISN1 harboring the *gusA-intron* [40] was transformed into an *A. tumefaciens* strain with virulence gene expression induced by acetosyringone (AS) before infection of 4-day-old seedlings. The seedlings were co-cultured with pre-induced *A. tumefaciens* in the presence of AS, and GUS activity was determined to monitor transient transformation efficiency at 3 days post-infection (dpi). Remarkably, all five T-pilus^−^/Vir^+^ uncoupling mutants showed highly reduced GUS stains in cotyledons with 3- to 4-fold lower GUS activity as compared to the wild type (Figure 5A and 5C). In contrast, five ExB2^+^/Vir^+^ mutants exhibited higher transient transformation efficiency than all T-pilus^−^/Vir^+^ uncoupling mutants, four showing efficiency comparable to that of the wild type (Figure 5B and C). These results suggest the importance of T-pilus in the *Agrobacterium* transformation process.

### Transient transformation assays of *Arabidopsis* seedlings with wounded cotyledons

**Figure 6:**
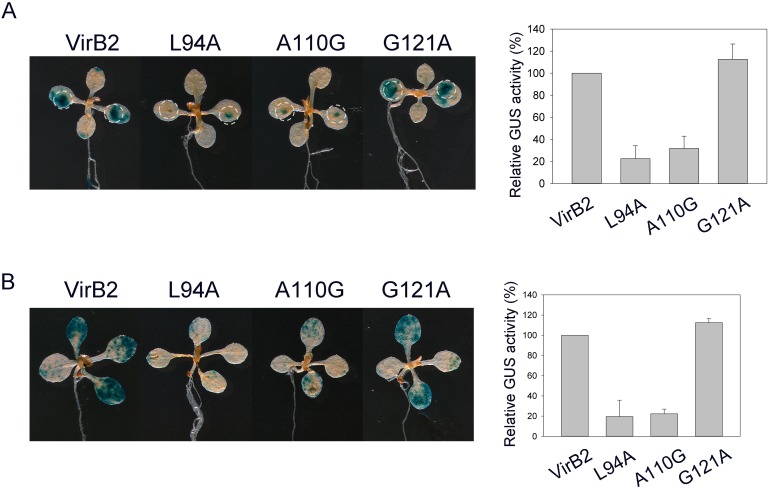
Transient transformation assay in *Arabidopsis* seedlings with or without wounded cotyledons. *A. tumefaciens* strains expressing the wild type or variants of VirB2, L94A (T-pilus^−^/Vir^+^), A110G (T-pilus^−^/Vir^+^) and G121A (T-pilus^+^/Vir^+^), harboring the T-DNA vector pBISN1 were used to infect 7-day-old *Arabidopsis* seedlings. GUS activity as a reporter of transient transformation efficiency was determined by GUS staining or quantitative activity assay at 3 dpi. (A) Infection of *Arabidopsis* seedlings with cotyledons wounded by a needle before infection. *A. tumefaciens* cells grown in 523 overnight culture without AS induction were used to infect the wounded *Arabidopsis* seedlings in the absence of AS. (B) Infection of *Arabidopsis* seedlings without intentional wounding, in which *A. tumefaciens* cells were pre-induced by AS for infection and co-cultured in the presence of AS. Data for quantitative GUS activity are mean±SD of four biological repeats from two independent experiments (10 seedlings in each biological repeat).

In contrast to tumor assays on tomato stems and potato tuber discs, with *A. tumefaciens* cells infected on wounded tissues, no intentional wounding was included during *A. tumefaciens* infection in *Arabidopsis* seedlings. Thus, we tested whether the T-pilus^−^/Vir^+^ uncoupling mutants could efficiently infect wounded tissue of *Arabidopsis* seedlings. Because cotyledons are highly transformed in the *Arabidopsis* seedling transient transformation assay, we wounded cotyledons of 7-day-old *Arabidopsis* seedlings by using a needle. Similar to the method used to infect wounded tomato stems and potato tuber discs, overnight-grown *A. tumefaciens* cells without AS induction were used to infect wounded cotyledons of *Arabidopsis* seedlings. As shown in Figure 6A, we detected large GUS stains extending from the wound site of cotyledons on co-culture with cells containing wild-type VirB2 or the ExB2^+^/Vir^+^ G121A variant, which also revealed wild-type tumorigeneisis efficiency in potato tuber discs (Figure S3) and transient transformation efficiency in intact *Arabidopsis* seedlings (Figure 5A and C). In contrast, GUS signals were detected only at a focused wound site of cotyledons and not expanded to unwounded regions on co-culture with the two T-pilus^−^/Vir^+^ uncoupling mutants tested (L94A and A110G). As a control, 7-day-old seedlings were infected with pre-induced *A. tumefaciens* cells under unwounded conditions (Figure 6B). We detected 3- to 4- fold lower GUS activity in the uncoupling mutants as compared with *A. tumefaciens* cells producing wild-type VirB2 or the ExB2^+^/Vir^+^ G121A variant, which efficiently infects both cotyledons and newly emerged true leaves not restricted to specific regions (Figure 6A and B). Because T-DNA transient transformation activity does not require the step(s) of T-DNA integration into the plant genome [40], T-pilus may play a role in the T-DNA transfer process at steps before T-DNA integration when infecting wounded and unwounded cotyledons of *Arabidopsis* seedlings.

## Discussion

In this study, we identified the VirB2 key amino acid residues involved in VirB2 stability, extracellular VirB2/T-pilus production, and virulence of *A. tumefaciens* and discovered a role for the T-pilus in enhancing transient transformation efficiency. By screening 34 VirB2 variants for their ability to promote T-pilus production and tumorigenesis, we found that all mutants that are capable of producing extracellular VirB2 are virulent, and all mutants with loss of virulence (no tumor) also do not produce extracellular VirB2. Because no mutants with the ExB2^+^/Vir^−^ phenotype could be isolated in our screen, the VirB2 amino acid residue essential for forming a functional T4SS for substrate translocation may also be required for production of extracellular VirB2 and/or T-pilus.

Interestingly, the G119A variant generated in this study showed low amounts of extracellular VirB2 and full virulence phenotypes (Figure 2), whereas the substitution of Glycine 119 with Cysteine in pTiA6 VirB2 caused loss of virulence but retained function in producing wild-type levels of extracellular VirB2 and T-pilus [22]. To clarify the phenotype discrepancy between pTiC58 G119A and pTiA6 G119C, we generated the pTiC58 VirB2 G119C amino acid substitution as for 34 other VirB2 variants and expressed it in C58 Δ*virB2*. By examining the levels of extracellular VirB2 in strains producing wild-type VirB2 and the G119A and G119C variants in parallel, all three independent G119C variants and the G119A variant produced low levels of extracellular VirB2 as compared with wild-type VirB2 (Figure S4). However, by examining all three independent pTiC58 G119C variants in three independent experiments, we did not observe any rigid and long T-pilus-like filament with ∼10 nm diameter. In contrast, we detected the presence of T-pilus produced with the pTiC58 G119A variant, although the number of T-pilus observed was less frequent than with the wild-type VirB2 (Figure 4).

Transient transformation efficiency was comparable with the G119A variant and the wild type VirB2, but no transient GUS activity was detected in *Arabidopsis* seedlings infected with the G119C variant (Figure S5). Consistently, the G119A variant also retained the wild-type tumorigenicity in tomato stems and potato tuber discs, whereas the G119C variant showed highly attenuated induction of tumors on potato tuber discs (Figure S5A). Since G119A and G119C variants both caused reduced or abolished extracellular VirB2/T-pilus production, Glycine 119 may be critical for the efficient assembly of T-pilin into extracellular T-pilus. The nonpolar amino acid at position 119 seems critical for efficient T-DNA and/or effector translocation because replacing nonpolar Glycine 119 with polar Cysteine but not nonpolar Alanine strongly suppressed the tumorigenesis/transformation efficiency. Although the loss of extracellular VirB2 in both S1 and S2 fractions is consistent with the lack of any observable long and rigid T-pilus-like filament in our five T-pilus^−^/Vir^+^ uncoupling mutants, these methods may have limitations to detect short T-pilus produced on bacterial cell surfaces. It is also possible that certain VirB2 variants may assemble T-pilus only during infection *in planta.* To this end, our data revealed the positive correlation of long and rigid T-pilus with the ability to induce high transient transformation efficiency in *Arabidopsis,* although we observed no dose-dependent correlation. This result highlighted a critical role of T-pilus involved in transient transformation efficiency in *Arabidopsis* seedlings, but T-pilus seems to be dispensable for inciting tumors in wounded plant tissues. Consistently, the T-pilus^−^/Vir^+^ uncoupling mutants were isolated from amino acid substitution in other T4SS components including VirB6, VirB9, VirB10, and VirB11 [14], [32]–[36].

The lack of T-pilus with the pTiC58 G119C variant is in contrast to the abundant T-pilus production from the pTiA6 G119C variant, which is also defective in substrate transfer based on the deficiency in IncQ plasmid transfer and tumorigeneisis on the wound site of *Kalanchoe daigremontiana* leaves [22]. However, all the Cysteine-substitution VirB2 variants created by Kerr and Christie [22] were generated in a VirB2 C64S (Cysteine 64 substitution by Serine) background to perform Cysteine labeling for mapping the VirB2 membrane topology. Although pTiA6 C64S variant exhibited near-wild-type substrate translocation frequency, whether the T-pilus^+^/Vir^−^ phenotype observed by the pTiA6 VirB2 C64S-G119C variant is solely caused by G119C substitution remains to be determined. Thus, the difference in T-pilus production phenotypes between pTiC58 and pTiA6 VirB2 G119C variants could be caused by this additional C64S mutation created in pTiA6. In addition, another four pTiC58 VirB2 Alanine substitution variants showed contrasting phenotypes in virulence or extracellular VirB2/T-pilus production as compared with pTiA6 VirB2 Cysteine substitution variants (Table S4). Thus, future work by creating the VirB2 single amino acid substitution with identical amino acid is required to confirm the phenotype discrepancy observed between two Ti plasmids. Replacing a specific amino acid residue by an amino acid with both similar and opposite biochemical features may be critical to unambiguously identify the role of each specific amino acid in contributing the observed phenotype.

Of note, pre-induction of *A. tumefaciens vir* gene expression before infection is critical for successful transient transformation in intact *Arabidopsis* seedlings without intentional wounding (data not shown). In contrast, this *vir* pre-induction is not required when infecting wounded cotyledons of *Arabidopsis* seedlings (Figure 6A). Since T-pilus^−^/Vir^+^ uncoupling mutants caused reduced transient transformation efficiency when infecting *Arabidopsis* seedlings with wounded or unwounded cotyledons, T-pilus may contribute to enhance *Agrobacterium* transient transformation efficiency at both wounded and unwounded infection conditions. However, in contrast to the more pronounced GUS activity detected further from wound sites of cotyledons infected with T-pilus^+^-producing strains, GUS signals were detected only at a focused wound site of cotyledons infected with the T-pilus^−^/Vir^+^ uncoupling mutants. Thus, T-pilus may be dispensable for infection at a wound site but critical for infecting unwounded tissues/cells. This possibility may also explain why these T-pilus^−^/Vir^+^ uncoupling mutants retained their ability to incite tumor formation when infecting wounded tomato stems and/or potato tuber discs in this study and in wounded *K. daigremontiana* leaves in other studies [14], [32]–[36].

In summary, this study provides compelling evidence for a role of T-pilus in the *Agrobacterium* transformation process, although the mechanisms involved remain unclear. Recent microscopy studies revealed the formation of T4SS helical array around the bacterial cell and observed a pilus-like structure connecting bacterial cells to the plant cell [24], [25]. However, the Ti plasmid and T-pilus were found not required for *A. tumefaciens* attachment to the plant cell [41]. Thus, T-pilus may modulate plant responses to achieve optimal transformation efficiency. VirB5 was found localized in the T-pilus tip [42] and extracellular VirB5 can accelerate *Agrobacterium* transformation efficiency [43]. T-pilus may provide a vehicle to localize VirB5 or other substrates to the correct location and exert their functions on/in plants or the T-pilus/VirB2 may play a direct role. The exact role and molecular mechanisms underlying how T-pilus contributes to *Agrobacterium* transformation process await future investigation.

## Materials and Methods

### Bacterial strains, growth and T-pilus induction conditions

Bacterial strains and plasmids used are in Table S2. *A. tumefaciens* and *E. coli* were grown in 523 medium [44] at 28°C and LB at 37°C, respectively, with appropriate antibiotics. The plasmids were maintained by the addition of 50 µg/ml gentamycin (Gm) and 20 µg/ml kanamycin (Km) for *A. tumefaciens* and 20 µg/ml Km and 50 µg/ml Gm for *E. coli.* The T-pilus induction condition was as described [7]. Briefly, overnight culture of *A. tumefaciens* cells grown in 523 medium with appropriate antibiotics [44] were harvested and resuspended in liquid AB-MES minimal medium, pH 5.5 [7], with an adjusted OD_600_ of 0.1 for 4 hr without antibiotics. In total, 500 µl bacterial suspension was spreaded onto solid AB-MES medium with 200 µg/ml acetosyringone (AS) in a 150-mm Petri dish and incubated at 19°C for 3 days without antibiotics.

### Construction of mutant strains and complementing plasmids

Gene replacement with the suicide plasmid pJQ200KS to generate the *virB2* in-frame deletion mutant (deletion of amino acid residues 4 to 113) followed a previous study [45]. For construction of pJQ-*virB2* for gene replacement, *virB2*-up (550-bp) and *virB2*-down (720-bp) DNA fragments were amplified by *pfu* polymerase and digested with SacI/SpeI and SpeI/XhoI separately and ligated into pJQ200KS at SacI/XhoI sites. For generating a plasmid to express *virB2* for complementation (pVirB2), a 2.1-kb fragment the *virB* promoter and *virB1*, *virB2* and *virB3* genes (*virB*p-*B1*-*B2*-*B3*) was PCR-amplified from the *A. tumefaciens* C58 genome and ligated into pRL662 at SpeI/XhoI sites. For generating *virB2* mutants with single amino acid substitutions, corresponding primers in Table S3 were used to amplify mutated sequence templates, followed by further amplification by the universal primer pair VirBp-B1-SpeI-F and VirB3-XhoI-R to generate the 2.1-kb fragment for ligation into pRL662 at SpeI/XhoI sites. Sequences of the entire 2.1-kb fragment (*virB*p-*B1*-*B2*-*B3*) were confirmed by DNA sequencing to ensure that no additional mutations occurred via PCR.

### Isolation of intracellular and extracellular fractions

The procedure to isolate extracellular fractions was as described previously [7], with minor modifications. *A. tumefaciens* cells were scraped off a 150-mm AB-MES, pH 5.5, agar plate by adding 2 ml buffer A (10 mM phosphate buffer, pH 5.3); the resulting cell suspension was centrifuged (13000×g, 4°C, 10 min) to collect the supernatant, named the S1 fraction. The resulting pellet was resuspended again in buffer A to OD_600_ 10 and divided into 1-ml aliquots. The cells were sheared through a 26-g needle syringe five times and harvested by centrifugation (13000×g, 4°C, 10 min) to collect the supernatant containing sheared T-pilus, named the S2 fraction. Both S1 and S2 fractions were collected and filtrated through a 0.22-µm low-protein-binding membrane (Minisart RC 15, Sartorius stedim biotech) to remove contaminating bacterial cells and precipitated by trichloroacetic acid (TCA) as described previously [45]. The sheared pellet was normalized to OD_600_ 10 and designated as the intracellular fraction.

### Tumor assay on tomato stems

Tomato cultivar FARMERS 301 from KNOWN-YOU SEED CO. (Kaohsiung, Taiwan) was grown at 23°C with a 16-/8-hr light/dark cycle. Two- to 3-week-old seedlings were wounded by use of a needle and inoculated with 5 µl *A. tumefaciens* cell suspension prepared as follows. *A. tumefaciens* cells were grown on a 523 agar plate at 28°C for 48 hr. Freshly grown colonies were re-suspended in 0.9% sodium chloride and adjusted to 10^8^ and 10^6^ CFU/ml for inoculation. Tumors were observed about 4 to 5 weeks after inoculation.

### SDS-PAGE and western blot analysis

Proteins were separated by 12% or 16% tricine SDS-PAGE [46] followed by western blot analysis as described [45]. For each protein sample, an equivalent number of cells was mixed with an equal volume of 2x SDS-PAGE loading buffer (0.1 M Tris-Cl, pH 6.8, 4% SDS, 0.1% bromophenol blue, 20% glycerol, 200 mM dithiothreitol) and incubated at 100°C for 10 min before loading. Polyclonal antisera VirB2-B23 (against the N-terminal region of processed VirB2 T-pilin encoded by pTiC58), VirB2-B24 (against the C-terminal region of processed VirB2 T-pilin encoded by pTiC58) [47] and RNA polymerase α-subunit RpoA [48] were used as primary antibodies. Horseradish peroxidase-conjugated goat anti-rabbit immunoglobulin G (Chemichem) was the secondary antibody and chemiluminescence was detected by the Western Lightning system (Perkin Elmer, Boston, MA) with X-ray film (Amersham).

### Electron microscopy

Procedures for negative staining were as described previously [6] with minor modifications. Briefly, *A. tumefaciens* cells grown under T-pilus induction conditions were collected, washed with pure water and re-suspend in 10 mM Tris buffer at pH 7.5. The bacterial suspension was deposited on a copper grid with carbon-Formvar film support for 1 min, rinsed with pure water for a few seconds, and stained with 2% uranyl acetate for 1 min. The samples were examined under a PHILIPS-CM100 transmission electron microscope (TEM) at 80 kV.

### Transient transformation assay in *Arabidopsis* seedlings

The method for transient transformation assay in *Arabidopsis* seedlings was as described [39]. In brief, *Arabidopsis thaliana* mutant *efr-1* lacking the elongation factor Tu (EF-Tu) receptor (SALK_044334) was used. Seeds were sterilized in 50% bleach and 0.05% Trition X-100 for 10 min and washed with sterile water five times and incubated in a 4°C refrigerator for 3 days before germination. Seeds were germinated in 1-ml MS liquid medium (1/2 MS salts, 0.5% sucrose, pH 5.7) in a 6-well plate (10 seedlings in each well) at 22°C, 16-/8-hr light/dark cycle for 4 or 7 days (indicated as 4- or 7-day-old seedlings). For *A. tumefaciens vir* gene pre-induction and infection, overnight cultured *A. tumefaciens* cells were re-suspended to OD_600_ 0.2 in AB-MES, pH 5.5, with 200 µM AS for growth at 28°C for 14 to 16 hr. Cells were re-suspended in infection medium (1/2 MS salts, 0.5% sucrose, 50 µM AS, pH 5.7) and adjusted to OD_600_ 0.02 and co-cultured with *Arabidopsis* seedlings at 22°C for 3 days. After co-cultivation, seedlings were stained with X-Gluc staining solution for 6 to 12 hr at 37°C or GUS activity was measured according to the *Arabidopsis* protocol [49].

### Tumor assay on potato tuber discs

Potato tumor assay was performed as described [45]. Briefly, overnight cultured *A. tumefaciens* cells were sub-cultured by 10X dilution and grown at 28°C to OD_600_ 1.0. Cells were washed with 0.9% sodium chloride and re-suspended in 0.9% sodium chloride at 10^8^ and 10^6^ CFU/ml. A total of 40 to 60 potato tuber disks were placed on water agar with each potato tuber disk infected with 10 µl bacterial culture and incubated at 22°C for 2 days. Disks were then placed on water agar supplemented with 100 µg/ml Timentin and tumors were scored with number of tumors/disc after incubation at 22°C for 3 to 4 weeks.

## Supporting Information

Figure S1 **Western blot analysis of the extracellular S2 fraction showing both high and low intensity.** *A. tumefaciens* cells grown on AS-induced AB-MES (pH 5.5) agar at 19°C for 3 days [7] were collected to isolate the extracellular S2 fractions. C58, *A. tumefaciens* wild type strain; V, empty vector pRL662; Δ*virB2*, *virB2* deletion mutant; Δ*virB2*(pVirB2), expression of wild type *virB*p-*B1*-*B2*-*B3* in Δ*virB2*. Western blot analysis with antisera against VirB2 B24 peptide or B23 peptide (for variants in C-PP) or RNA polymerase RpoA, as an internal control. Unprocessed VirB2 precursor is indicated as VirB2p and processed mature VirB2 as VirB2m. Each region/domain of VirB2 is indicated as described in Figure 1. Western blot images with high (longer exposure time) and low intensity (shorter exposure time) are shown.
(TIF)

Figure S2 **Western blot analysis of S1 fraction.** *A. tumefaciens* cells grown on AS-induced AB-MES (pH 5.5) agar at 19°C for 3 days [7] were collected to isolate intracellular proteins and extracellular S1 fraction. C58, *A. tumefaciens* wild type strain; V, empty vector pRL662; Δ*virB2*, *virB2* deletion mutant; Δ*virB2*(pVirB2), expression of wild type *virB*p-*B1*-*B2*-*B3* in Δ*virB2*. Western blot analysis with antisera against VirB2 B24 peptide or RNA polymerase RpoA, as an internal control. Processed mature VirB2 as VirB2m.
(TIF)

Figure S3 **Potato tumor assay of** ***A. tumefaciens*** **strains expressing wild-type VirB2 or variants of L94A (T-pilus^−^**/**Vir^+^), A110G (T-pilus^−^**/**Vir^+^) and G121A (T-pilus^+^/Vir^+^).** *A. tumefaciens* cells at 10^8^ and 10^6^ CFU/ml were used for infection. The potato tuber disks were placed on water agar, infected with 10 µl of bacterial cultures, and incubated at 22°C for 2 days. Disks were placed on water agar supplemented with 100 µg/ml Timentin and incubated at 22°C. Tumors were scored after 3 weeks. Data are mean±SEM of number of tumors averaged from 40–60 disks. Similar results were obtained from at least two independent experiments.
(TIF)

Figure S4 **Western blot analysis of the intracellular and extracellular S1 and S2 fractions of** ***A. tumefaciens*** **strains expressing wild-type VirB2, G119A, or G119C variants.** *A. tumefaciens* cells grown on AS-induced AB-MES (pH 5.5) agar at 19°C for 3 days [7] were collected to isolate the intracellular and extracellular S1 and S2 fractions. *A. tumefaciens* strain producing wild-type VirB2, G119A variant, and three independent colonies of G119C variant (G119C-1,-2 or -3) were analyzed. Western blot analysis with antisera against VirB2 B23 peptide or RNA polymerase RpoA, as an internal control. Processed mature VirB2 is indicated as VirB2m.
(TIF)

Figure S5 **Tumorigenesis and transient transformation assays of** ***A. tumefaciens*** **strains expressing wild-type VirB2, G119A, or G119C variants.** (A) Potato tumor assay. *A. tumefaciens* cells at 10^8^ and 10^6^ CFU/ml were used for infection. The potato tuber disks were placed on water agar, infected with 10 µgl of bacterial cultures, and incubated at 22°C for 2 days. Disks were placed on water agar supplemented with 100 µg/ml Timentin and incubated at 22°C. Tumors were scored after 3 weeks. Data are mean±SEM of number of tumors averaged from 40–60 disks. Similar results were obtained from at least two independent experiments. (B) Transient transformation assay in *Arabidopsis* seedlings. *A. tumefaciens* strains expressing wild-type or variants of VirB2 harboring T-DNA vector pBISN1 were used to infect 4-day-old *Arabidopsis* seedlings. GUS activity as a reporter for transient transformation efficiency was determined by GUS staining or quantitative activity assay at 3 dpi. Data for quantitative GUS activity are mean±SD of four biological repeats from two independent experiments (10 seedlings in each biological repeat).
(TIF)

Table S1 **Amino acid sequence homology analysis of selected VirB2 family proteins.**
(PDF)

Table S2 **Bacterial strains and plasmids.**
(DOCX)

Table S3 **Primer list.**
(PDF)

Table S4 **Phenotype comparisons of pTiC58 and pTiA6 VirB2 variants.**
(PDF)
