# Supplementary figures and images for: Expression and Functional Characterization of the Agrobacterium VirB2 Amino Acid Substitution Variants in T-pilus Biogenesis, Virulence, and Transient Transformation Efficiency
Source: PLoS One. 2014 Jun 27;9(6):e101142. doi: 10.1371/journal.pone.0101142 (PMC4074166; doi:10.1371/journal.pone.0101142)

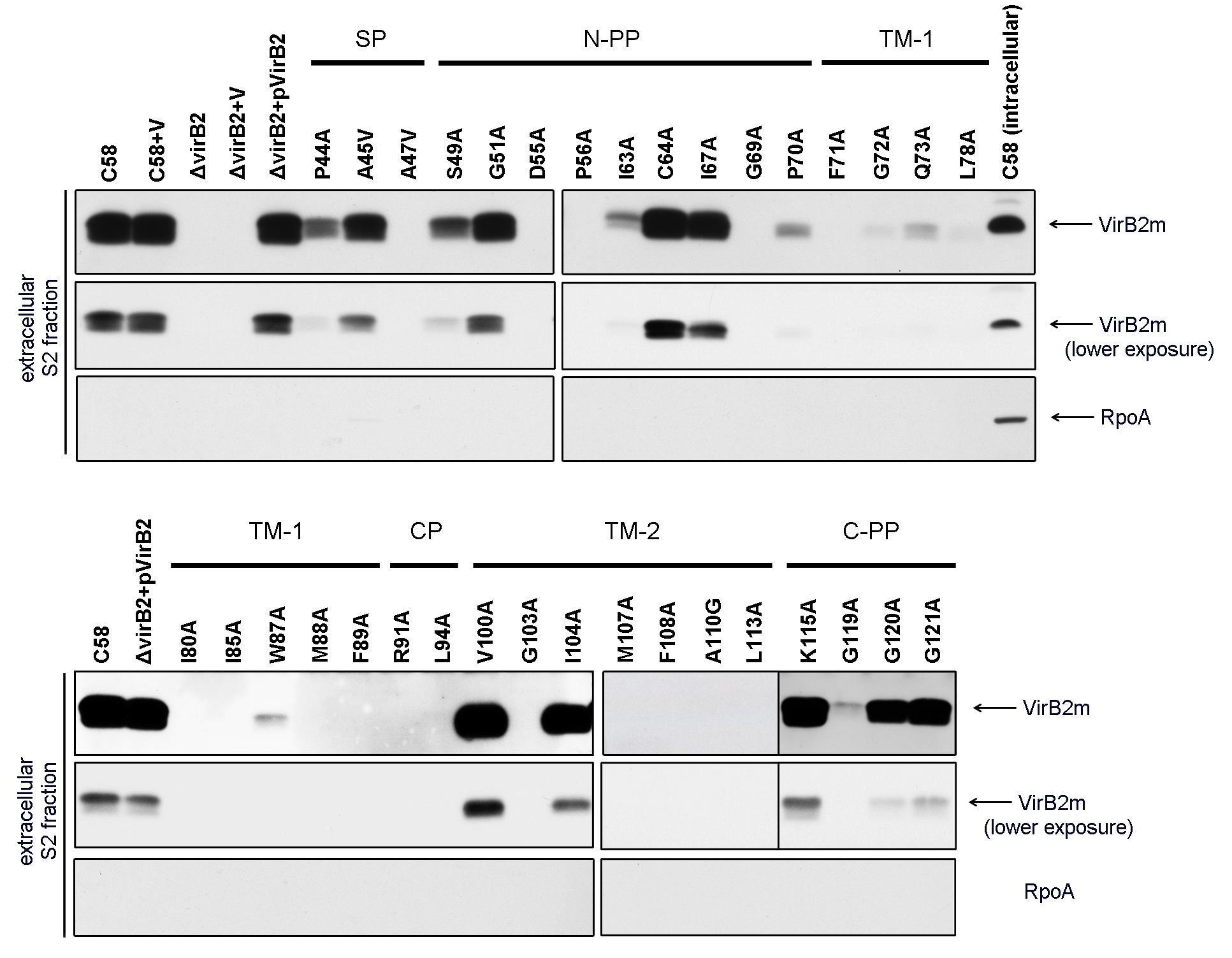

Supplement: Figure S1 — Western blot analysis of the extracellular S2 fraction showing both high and low intensity. A. tumefaciens cells grown on AS-induced AB-MES (pH 5.5) agar at 19°C for 3 days [7] were collected to isolate the extracellular S2 fractions. C58, A. tumefaciens wild type strain; V, empty vector pRL662; ΔvirB2, virB2 deletion mutant; ΔvirB2(pVirB2), expression of wild type virBp-B1-B2-B3 in ΔvirB2. Western blot analysis with antisera against VirB2 B24 peptide or B23 peptide (for variants in C-PP) or RNA polymerase RpoA, as an internal control. Unprocessed VirB2 precursor is indicated as VirB2p and processed mature VirB2 as VirB2m. Each region/domain of VirB2 is indicated as described in Figure 1. Western blot images with high (longer exposure time) and low intensity (shorter exposure time) are shown. (TIF) [file pone.0101142.s001.tif]

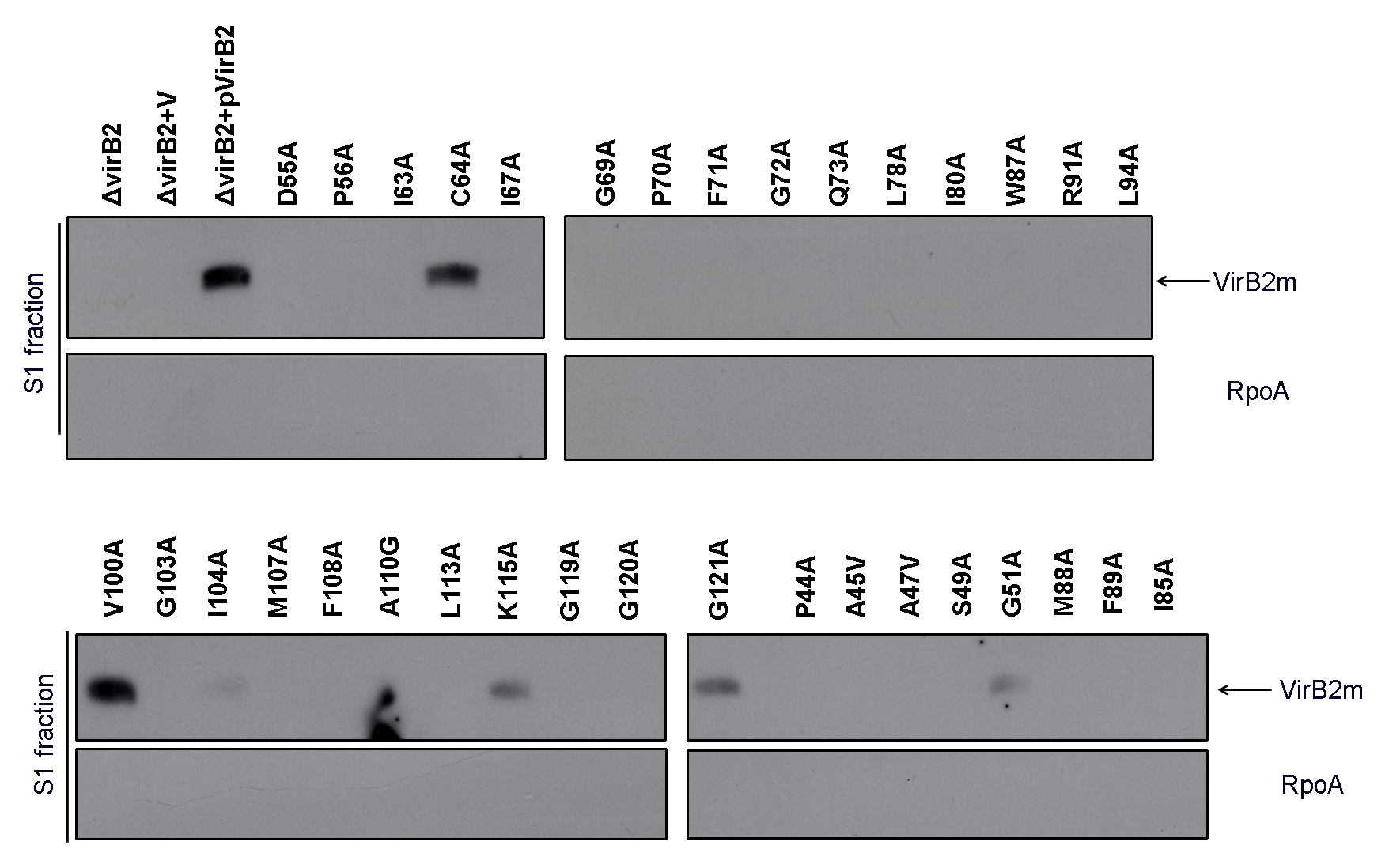

Supplement: Figure S2 — Western blot analysis of S1 fraction. A. tumefaciens cells grown on AS-induced AB-MES (pH 5.5) agar at 19°C for 3 days [7] were collected to isolate intracellular proteins and extracellular S1 fraction. C58, A. tumefaciens wild type strain; V, empty vector pRL662; ΔvirB2, virB2 deletion mutant; ΔvirB2(pVirB2), expression of wild type virBp-B1-B2-B3 in ΔvirB2. Western blot analysis with antisera against VirB2 B24 peptide or RNA polymerase RpoA, as an internal control. Processed mature VirB2 as VirB2m. (TIF) [file pone.0101142.s002.tif]

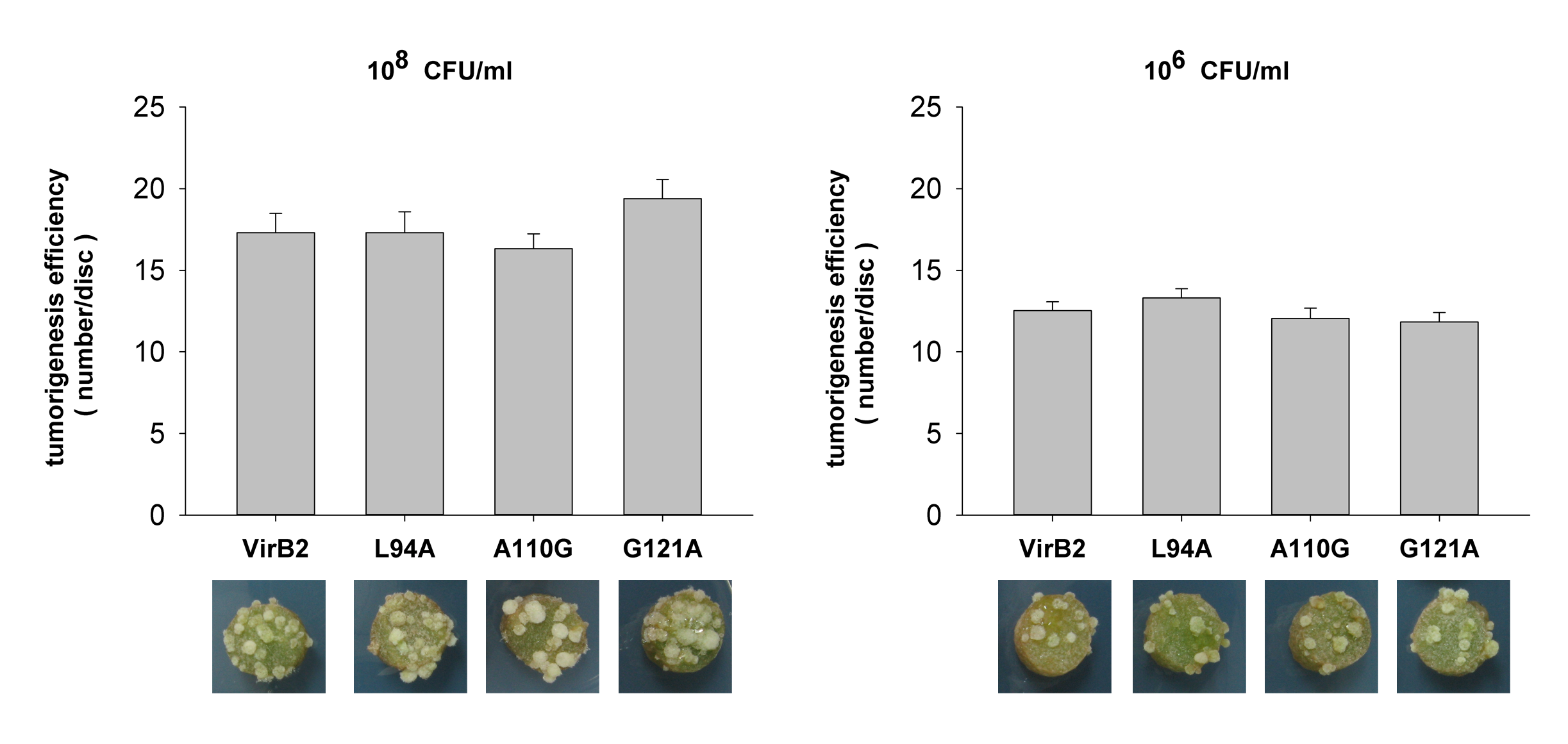

Supplement: Figure S3 — Potato tumor assay of A. tumefaciens strains expressing wild-type VirB2 or variants of L94A (T-pilus−/Vir+), A110G (T-pilus−/Vir+) and G121A (T-pilus+/Vir+). A. tumefaciens cells at 108 and 106 CFU/ml were used for infection. The potato tuber disks were placed on water agar, infected with 10 µl of bacterial cultures, and incubated at 22°C for 2 days. Disks were placed on water agar supplemented with 100 µg/ml Timentin and incubated at 22°C. Tumors were scored after 3 weeks. Data are mean±SEM of number of tumors averaged from 40–60 disks. Similar results were obtained from at least two independent experiments. (TIF) [file pone.0101142.s003.tif]

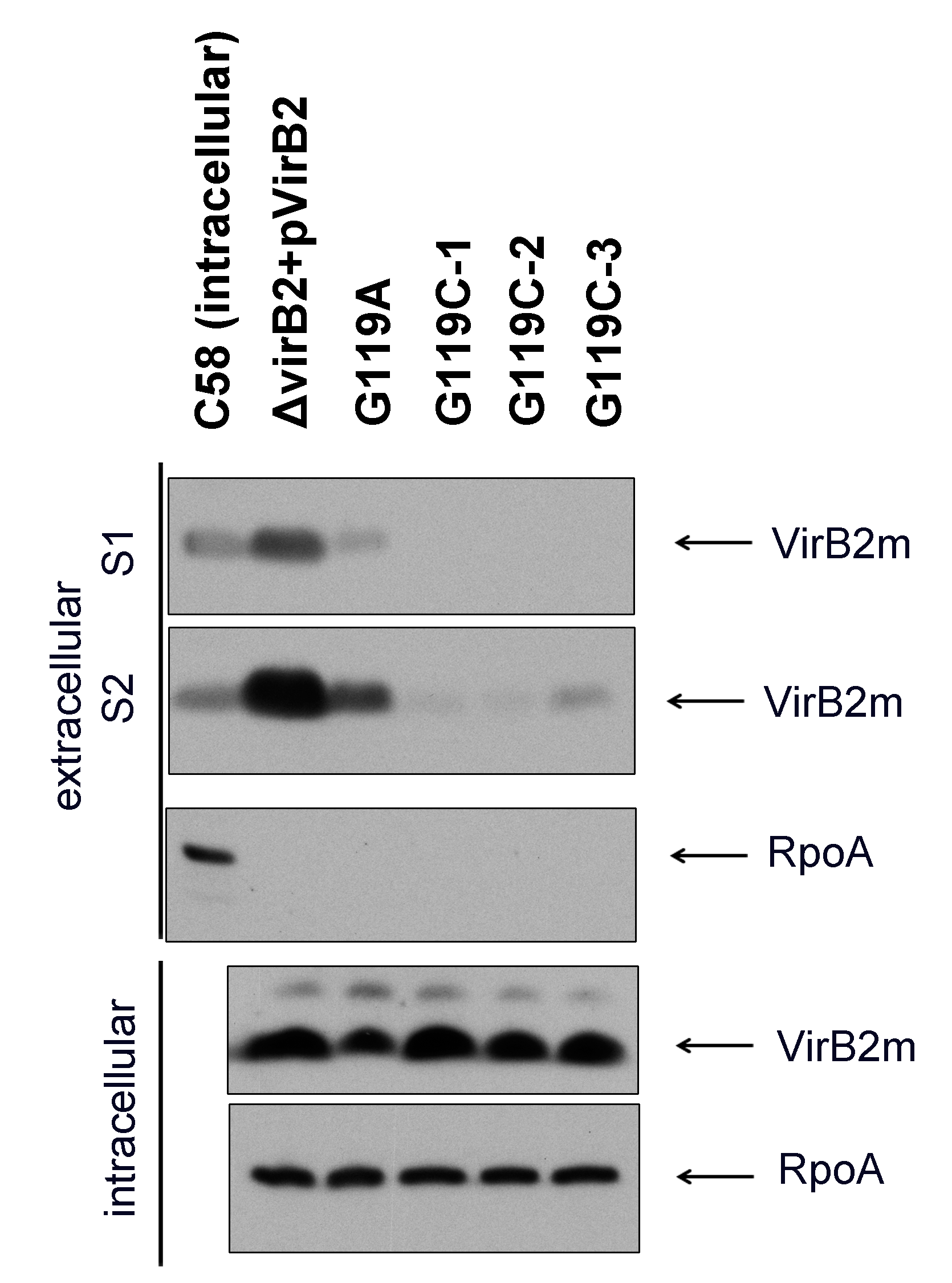

Supplement: Figure S4 — Western blot analysis of the intracellular and extracellular S1 and S2 fractions of A. tumefaciens strains expressing wild-type VirB2, G119A, or G119C variants. A. tumefaciens cells grown on AS-induced AB-MES (pH 5.5) agar at 19°C for 3 days [7] were collected to isolate the intracellular and extracellular S1 and S2 fractions. A. tumefaciens strain producing wild-type VirB2, G119A variant, and three independent colonies of G119C variant (G119C-1,-2 or -3) were analyzed. Western blot analysis with antisera against VirB2 B23 peptide or RNA polymerase RpoA, as an internal control. Processed mature VirB2 is indicated as VirB2m. (TIF) [file pone.0101142.s004.tif]

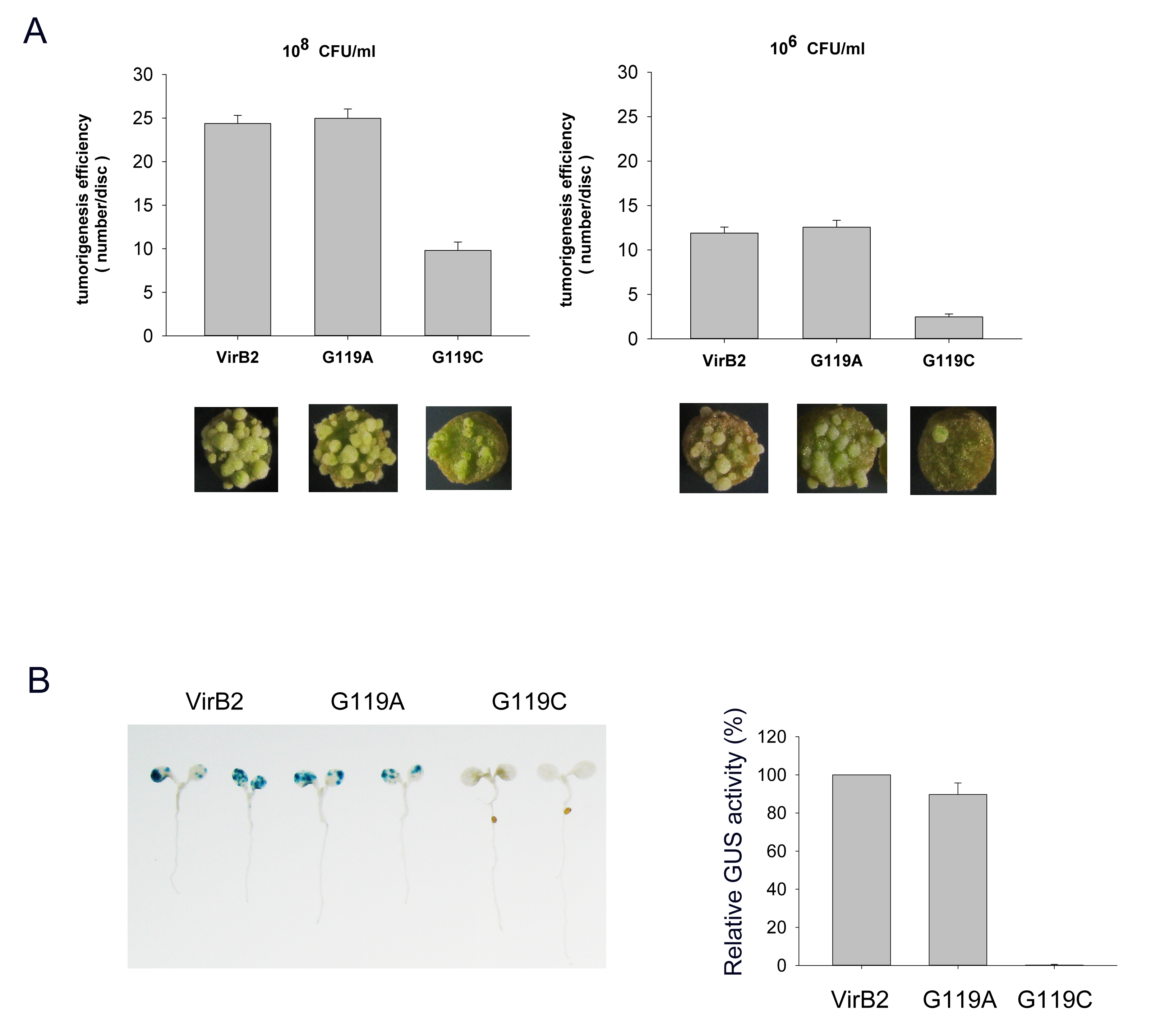

Supplement: Figure S5 — Tumorigenesis and transient transformation assays of A. tumefaciens strains expressing wild-type VirB2, G119A, or G119C variants. (A) Potato tumor assay. A. tumefaciens cells at 108 and 106 CFU/ml were used for infection. The potato tuber disks were placed on water agar, infected with 10 µgl of bacterial cultures, and incubated at 22°C for 2 days. Disks were placed on water agar supplemented with 100 µg/ml Timentin and incubated at 22°C. Tumors were scored after 3 weeks. Data are mean±SEM of number of tumors averaged from 40–60 disks. Similar results were obtained from at least two independent experiments. (B) Transient transformation assay in Arabidopsis seedlings. A. tumefaciens strains expressing wild-type or variants of VirB2 harboring T-DNA vector pBISN1 were used to infect 4-day-old Arabidopsis seedlings. GUS activity as a reporter for transient transformation efficiency was determined by GUS staining or quantitative activity assay at 3 dpi. Data for quantitative GUS activity are mean±SD of four biological repeats from two independent experiments (10 seedlings in each biological repeat). (TIF) [file pone.0101142.s005.tif]
