# Supplementary material for: Expression and Functional Characterization of the Agrobacterium VirB2 Amino Acid Substitution Variants in T-pilus Biogenesis, Virulence, and Transient Transformation Efficiency
Source: PLoS One. 2014 Jun 27;9(6):e101142. doi: 10.1371/journal.pone.0101142 (PMC4074166; doi:10.1371/journal.pone.0101142)
Supplement: Table S1 — Amino acid sequence homology analysis of selected VirB2 family proteins. (PDF) [file pone.0101142.s006.pdf]

Table S1 Amino acid sequence homology analysis of selected VirB2 family proteins.

Protein[a] name	*A. t.* C58 pTiC58 VirB2	*A. r.* pRi1724 Riorf154	*A. t.* pTiA6 VirB2	*A. v.* S4 pTiS4 VirB2	*R. e.* CFN42 p42a VirB2a	*S. m.* WSM419 pSMED02 VirB2	*M. c. bv. biserrulae* TrbC	*A. r.* K84 AvhB2	*A. t.* C58 pAtC58 AvhB2	*R. e.* CFN42 p42d VirB2	*S. m.* 1021 pSymA VirB2
VirB2p[b]	100 (100)	75 (81)	90 (95)	74 (82)	93 (98)	69 (76)	71 (77)	40 (56)	36 (56)	35 (56)	35 (50)
VirB2m[c]	100 (100)	96 (96)	99 (100)	96 (96)	95 (100)	93 (94)	91 (92)	44 (61)	35 (55)	38 (57)	35 (59)

[a]: Organism full name and protein UniProt accession number: *Agrobacterium tumefaciens* pTiC58, P17792; *Agrobacterium rhizogenes* pRi1724, Q9F5A1; *Agrobacterium tumefaciens* pTiA6, P05351; *Agrobacterium vitis* pTiS4, B9K417; *Rhizobium etli* p42a, Q2K2L1; *Sinorhizobium medicae* pSMED02; A6UMA7, *Mesorhizobium ciceri* chromosome, E8TGI0; *Agrobacterium radiobacter* K84 chromosome, B9JE70; *Agrobacterium tumefaciens* pAtC58, Q7D3S1; *Rhizobium etli* p42d**,** Q8KIM6; *Sinorhizobium meliloti* pSymA, Q92YZ4.

[b] identity % (similarity %) of full-length pTiC58 VirB2 against other VirB2 homologs.

[c] identity % (similarity %) of processed pTiC58 VirB2 against other VirB2 homologs.
