# Supplementary material for: Expression and Functional Characterization of the Agrobacterium VirB2 Amino Acid Substitution Variants in T-pilus Biogenesis, Virulence, and Transient Transformation Efficiency
Source: PLoS One. 2014 Jun 27;9(6):e101142. doi: 10.1371/journal.pone.0101142 (PMC4074166; doi:10.1371/journal.pone.0101142)
Supplement: Table S2 — Bacterial strains and plasmids. (DOCX) [file pone.0101142.s007.docx]

**Table S2 Bacterial strains and plasmids**

|  | Strain / plasmids | Related characteristics | Source |
| --- | --- | --- | --- |
| *A. tumefaciens* | | |  |
|  | C58 | Wild type virulent strain | [1] |
|  | C58Δ*virB2* | *virB2* in-frame deletion mutant | This study |
|  | C58Δ*virB2*+p*virB2* | Gm^R^, C58Δ*virB2* harbor pRL4 complementary vector | This study |
| Plasmids | |  |  |
|  | pRL662 | Gm^R^, Broad host range vector derived from pBBR1MCS-2 | [2] |
|  | pJQ200KS | Gm^R^, Suicide plasmid for double crossoer | [3] |
|  | pBISN1 | Km^R^, Binary vector expressing *gusA-intron* driven by super promoter | [4] |
|  | pJQ-VirB2 | Gm^R^, Vector used to generate *virB2* in-frame deletion mutant | This study |
|  | pRL4(p*virB2*) | Gm^R^, pRL662 vector expression *virB1-virB2-virB3* gene driven by native *virB* promoter | This study |
|  | p*virB2*-P44A | Gm^R^, pRL4 vector containing a *virB2* P44A substitution | This study |
|  | p*virB2*-A45V | Gm^R^, pRL4 vector containing a *virB2* A45V substitution | This study |
|  | p*virB2*-A47V | Gm^R^, pRL4 vector containing a *virB2* A47V substitution | This study |
|  | p*virB2*-S49A | Gm^R^, pRL4 vector containing a *virB2* S49A substitution | This study |
|  | p*virB2*-G51A | Gm^R^, pRL4 vector containing a *virB2* G51A substitution | This study |
|  | p*virB2*-D55A | Gm^R^, pRL4 vector containing a *virB2* D55A substitution | This study |
|  | p*virB2*-P56A | Gm^R^, pRL4 vector containing a *virB2* P56A substitution | This study |
|  | p*virB2*-I63A | Gm^R^, pRL4 vector containing a *virB2* I63A substitution | This study |
|  | p*virB2*-C64A | Gm^R^, pRL4 vector containing a *virB2* C64A substitution | This study |
|  | p*virB2*-I67A | Gm^R^, pRL4 vector containing a *virB2* I67A substitution | This study |
|  | p*virB2*-G69A | Gm^R^, pRL4 vector containing a *virB2* G69A substitution | This study |
|  | p*virB2*-P70A | Gm^R^, pRL4 vector containing a *virB2* P70A substitution | This study |
|  | p*virB2*-F71A | Gm^R^, pRL4 vector containing a *virB2* F71A substitution | This study |
|  | p*virB2*-G72A | Gm^R^, pRL4 vector containing a *virB2* G72A substitution | This study |
|  | p*virB2*-Q73A | Gm^R^, pRL4 vector containing a *virB2* Q73A substitution | This study |
|  | p*virB2*-L78A | Gm^R^, pRL4 vector containing a *virB2* L78A substitution | This study |
|  | p*virB2*-I80A | Gm^R^, pRL4 vector containing a *virB2* I80A substitution | This study |
|  | p*virB2*-I85A | Gm^R^, pRL4 vector containing a *virB2* I85A substitution | This study |
|  | p*virB2*-W87A | Gm^R^, pRL4 vector containing a *virB2* W87A substitution | This study |
|  | pv*irB2*-M88A | Gm^R^, pRL4 vector containing a *virB2* M88A substitution | This study |
|  | p*virB2*-F89A | Gm^R^, pRL4 vector containing a *virB2* F89A substitution | This study |
|  | p*virB2*-R91A | Gm^R^, pRL4 vector containing a *virB2* R91A substitution | This study |
|  | p*virB2*-L94A | Gm^R^, pRL4 vector containing a *virB2* L94A substitution | This study |
|  | p*virB2*-V100A | Gm^R^, pRL4 vector containing a *virB2* V100A substitution | This study |
|  | p*virB2*-G103A | Gm^R^, pRL4 vector containing a *virB2* G103A substitution | This study |
|  | p*virB2*-I104A | Gm^R^, pRL4 vector containing a *virB2* I104A substitution | This study |
|  | p*virB2*-M107A | Gm^R^, pRL4 vector containing a *virB2* M107A substitution | This study |
|  | p*virB2*-F108A | Gm^R^, pRL4 vector containing a *virB2* F108A substitution | This study |
|  | p*virB2*-A110G | Gm^R^, pRL4 vector containing a *virB2* A110G substitution | This study |
|  | p*virB2*-L113A | Gm^R^, pRL4 vector containing a *virB2* L113A substitution | This study |
|  | p*virB2*-K115A | Gm^R^, pRL4 vector containing a *virB2* K115A substitution | This study |
|  | p*virB2*-G119A | Gm^R^, pRL4 vector containing a *virB2* G119A substitution | This study |
|  | p*virB2*-G119C | Gm^R^, pRL4 vector containing a *virB2* G119C substitution | This study |
|  | p*virB2*-G120A | Gm^R^, pRL4 vector containing a *virB2* G120A substitution | This study |
|  | p*virB2*-G121A | Gm^R^, pRL4 vector containing a *virB2* G121A substitution | This study |

**References**

1. Hamilton RH, Fall MZ (1971) The loss of tumor-initiating ability in *Agrobacterium* *tumefaciens* by incubation at high temperature. Experientia 27: 229-230.

2. Vergunst AC, Schrammeijer B, den Dulk-Ras A, de Vlaam CM, Regensburg-Tuink TJ, et al. (2000) VirB/D4-dependent protein translocation from *Agrobacterium* into plant cells. Science 290: 979-982.

3. Quandt J, Hynes MF (1993) Versatile suicide vectors which allow direct selection for gene replacement in gram-negative bacteria. Gene 127: 15-21.

4. Narasimhulu SB, Deng XB, Sarria R, Gelvin SB (1996) Early transcription of *Agrobacterium* T-DNA genes in tobacco and maize. Plant Cell 8: 873-886.
