# Supplementary material for: Expression and Functional Characterization of the Agrobacterium VirB2 Amino Acid Substitution Variants in T-pilus Biogenesis, Virulence, and Transient Transformation Efficiency
Source: PLoS One. 2014 Jun 27;9(6):e101142. doi: 10.1371/journal.pone.0101142 (PMC4074166; doi:10.1371/journal.pone.0101142)
Supplement: Table S3 — Primer list. (PDF) [file pone.0101142.s008.pdf]

Table S3 primer list

Primer name	Sequence(5’→3’)	Related information
VirBp-B1-SpeI-F	CCCACTAGTCACTTTCCAGCGATGACATGA	Universal primers of pRL4 and single amino acid substitution mutants
VirB3-XhoI-R	TCTCCTCGAGTTACGCCATTCCTCTCCCT	
VirB2-up-SacI-sense	TTAAGAGCTCGCAGAACCAGGCTCAAGC	Generate *virB2*-up fragment for pJQ-*virB2*
VirB2-up-SpeI-anti	TCTACTAGTGCATCGCATTATTGCGGACCT	
VirB2-down-SpeI-sense	TAATACTAGTGGCAAAACGCTGACTGGAGG	Generate *virB2*-down fragment for pJQ-*virB2*
VirB2-dwon-XhoI-anti	TAAGCTCGAGGTCATTGCGAAGGAGTTT	
VirB2-P44A-Sense	GCATTTTCTCCTCTGGGGCAGCCGC	Generate VirB2-P44A
VirB2-P44A-Anti	GCGGCTGCCCCAGAGGAGAAAATGC	
VirB2-A45V-Sense	GCCGGTTGCGGCCCAATCTGCAGGT	Generate VirB2-A45V
VirB2-A45V- Anti	ACCTGCAGATTGGGCCGCAACCGGC	
VirB2-A47V-Sense	GCGGTCCAATCTGCAGGTGGCG	Generate VirB2-A47V
VirB2-A47V- Anti	CGCCACCTGCAGATTGGACCGC	
VirB2-S49A-Sense	GCCCAAGCAGCAGGTGGCGGCACTG	Generate VirB2-S49A
VirB2-S49A- Anti	CAGTGCCGCCACCTGCTGCTTGGGC	
VirB2-G51A-Sense	CCAATCTGCAGCTGGCGGCACTGAC	Generate VirB2-G51A
VirB2-G51A- Anti	GTCAGTGCCGCCAGCTGCAGATTGG	
VirB2-D55A-Sense	TGGCGGCACTGCCCCAGCCACAATG	Generate VirB2-D55A
VirB2-D55A- Anti	CATTGTGGCTGGGGCAGTGCCGCCA	
VirB2-P56A-Sense	GGCACTGACGCCGCCACAATGGTTA	Generate VirB2-P56A
VirB2-P56A- Anti	TAACCATTGTGGCGGCGTCAGTGCC	
VirB2-I63A-Sense	ACAATGGTTAACAACGCATGCACGTTTATC	Generate VirB2-I63A
VirB2-I63A- Anti	GATAAACGTGCATGCGTTGTTAACCATTGT	
VirB2-C64A-Sense	TTAACAACATAGCCACGTTTATCCT	Generate VirB2-C64A
VirB2-C64A-anti	AGGATAAACGTGGCTATGTTGTTAA	
VirB2-I67A-Sense	TATGCACGTTTGCCCTTGGTCCGTT	Generate VirB2-I67A
VirB2-I67A- Anti	AACGGACCAAGGGCAAACGTGCATA	
VirB2-G69A-Sense	CGTTTATCCTTGCTCCGTTCGGCCA	Generate VirB2-G69A
VirB2-G69A- Anti	TGGCCGAACGGAGCAAGGATAAACG	

VirB2-P70A-Sense	TTATCCTTGGTGCCTTCGGCCAGTC	Generate VirB2-P70A
VirB2-P70A- Anti	GACTGGCCGAAGGCACCAAGGATAA	
VirB2-F71A-Sense	TCCTTGGTCCGGCTGGCCAGTCACT	Generate VirB2-F71A
VirB2-F71A- Anti	AGTGACTGGCCAGCCGGACCAAGGA	
VirB2-G72A-Sense	TTGGTCCGTTCGCCCAGTCACTCGC	Generate VirB2-G72A
VirB2-G72A- Anti	GCGAGTGACTGGGCGAACGGACCAA	
VirB2-Q73A-Sense	GTCCGTTCGGCGCATCACTCGCTGTT	Generate VirB2-Q73A
VirB2-Q73A- Anti	AACAGCGAGTGATGCGCCGAACGGAC	
VirB2-L78A-Sense	CACTCGCTGTTGCTGGCATCGTGGC	Generate VirB2-L78A
VirB2-L78A- Anti	GCCACGATGCCAGCAACAGCGAGTG	
VirB2-I80A-Sense	CGCTGTTCTTGGCGCTGTGGCCATCGGAA	Generate VirB2-I80A
VirB2-I80A- Anti	TTCCGATGGCCACAGCGCCAAGAACAGCG	
VirB2-I85A-Sense	GCCATCGGAGCCTCCTGGATGTTCG	Generate VirB2-I85A
VirB2-I85A- Anti	CGAACATCCAGGAGGCTCCGATGGC	
VirB2-W87A-Sense	TCGGAATCTCCGCCATGTTCGGTCG	Generate VirB2-W87A
VirB2-W87A- Anti	CGACCGAACATGGCGGAGATTCCGA	
VirB2-M88A-Sense	CGGAATCTCCTGGGCTTTCGGTCGC	Generate VirB2-M88A
VirB2-M88A- Anti	GCGACCGAAAGCCCAGGAGATTCCG	
VirB2-F89A-Sense	AATCTCCTGGATGGCCGGTCGCGCT	Generate VirB2-F89A
VirB2-F89A- Anti	AGCGCGACCGGCCATCCAGGAGATT	
VirB2-R91A-Sense	CTGGATGTTCGGTGCTGCTTCACTCGGT	Generate VirB2-R91A
VirB2-R91A- Anti	ACCGAGTGAAGCAGCACCGAACATCCAG	
VirB2-L94A-Sense	GTCGCGCTTCAGCCGGTCTCGTTGC	Generate VirB2-L94A
VirB2-L94A- Anti	GCAACGAGACCGGCTGAAGCGCGAC	
VirB2-V100A-Sense	TCGTTGCCGGCGCAGTCGGCGGCAT	Generate VirB2-V100A
VirB2-V100A- Anti	ATGCCGCCGACTGCGCCGGCAACGA	
VirB2-G103A-Sense	GCGTCGTCGGCGCTATTGTCATCAT	Generate VirB2-G103A
VirB2-G103A- Anti	ATGATGACAATAGCGCCGACGACGC	
VirB2-I104A-Sense	TCGTCGGCGGCGCTGTCATCATGTT	Generate VirB2-I104A
VirB2-I104A- Anti	AACATGATGACAGCGCCGCCGACGA	
VirB2-M107A-Sense	GGCATTGTCATCGCTTTTGGAGCCAGC	Generate VirB2-M107A
VirB2-M107A- Anti	GCTGGCTCCAAAAGCGATGACAATGCC	
VirB2-F108A-Sense	ATTGTCATCATGGCTGGAGCCAGCTTCC	Generate VirB2-F108A
VirB2-F108A- Anti	GGAAGCTGGCTCCAGCCATGATGACAAT	
VirB2-A110G-Sense	TCATGTTTGGAGGCAGCTTCCTGGG	Generate VirB2-A110G
VirB2-A110G- Anti	CCCAGGAAGCTGCCTCCAAACATGA	
VirB2-L113A-Sense	GAGCCAGCTTCGCAGGCAAAACGCT	Generate

VirB2-L113A- Anti	AGCGTTTTGCCTGCGAAGCTGGCTC	VirB2-L113A
VirB2-K115A-Sense	GCTTCCTGGGCGCAACGCTGACTGG	Generate
VirB2-K115A- Anti	CCAGTCAGCGTTGCGCCCAGGAAGC	VirB2-K115A
VirB2-G119A-Sense	CAAAACGCTGACTGCTGGTGGCTAATGA	Generate
VirB2-G119A- Anti	TCATTAGCCACCAGCAGTCAGCGTTTTG	VirB2-G119A
VirB2-G119C-Sense	CAAAACGCTGACTTGCGGTGGCTAATGA	Generate
VirB2-G119C- Anti	TCATTAGCCACCGCAAGTCAGCGTTTTG	VirB2-G119C
VirB2-G120A-Sense	ACGCTGACTGGAGCTGGCTAATGAATGA	Generate
VirB2-G120A- Anti	TCATTCATTAGCCAGCTCCAGTCAGCGT	VirB2-G120A
VirB2-G121A-Sense	CTGACTGGAGGTGCTTAATGAATGATCG	Generate
VirB2-G121A- Anti	CGATCATTCATTAAGCACCTCCAGTCAG	VirB2-G121A
