# Supplementary material for: Expression and Functional Characterization of the Agrobacterium VirB2 Amino Acid Substitution Variants in T-pilus Biogenesis, Virulence, and Transient Transformation Efficiency
Source: PLoS One. 2014 Jun 27;9(6):e101142. doi: 10.1371/journal.pone.0101142 (PMC4074166; doi:10.1371/journal.pone.0101142)
Supplement: Table S4 — Phenotype comparisons of pTiC58 and pTiA6 VirB2 variants. (PDF) [file pone.0101142.s009.pdf]

Table S4 Phenotype comparisons of pTiC58 and pTiA6 VirB2 variants

Amino acid residue	Substitution	Ti-plasmid	Extracellular VirB2[a]	Virulence[b]
G51	A	C58	+++	+++
	C	A6	-	-
C64	A	C58	+++	+++
	C	A6	+++	+++
I67	A	C58	+++	+++
	C	A6	+++	+++
F71	A	C58	-	-
	C	A6	+	-
I85	A	C58	-	+++
	C	A6	+++	+++
M88	A	C58	-	-
	C	A6	++	++
L94	A	C58	-	+++
	C	A6	+++	++
I104	A	C58	+++	+++
	C	A6	+++	+++
M107	A	C58	-	+++
	C	A6	+++	+++
G119	A	C58	+	+++
	C	C58	+	+
	C	A6	+++	-

The levels of extracellular VirB2 production[a] and occurrence or size of tumor formation on tomato stems[b] were were based on results from Figure 2, 4, and S5A in this study and previous study by Kerr and Christie (2010) [1]. The levels of extracellular VirB2 and virulence strength are indicated with wild type (+++), modest reduction (++), highly attenuation (+), or loss (-).

Reference

1. Kerr JE, Christie PJ (2010) Evidence for VirB4-Mediated Dislocation of Membrane-Integrated VirB2 Pilin During Biogenesis of the *Agrobacterium* VirB/VirD4 Type IV Secretion System. J Bacteriol. 192: 4923-34.
